# Effects of Sodium-Glucose Cotransporter 2 Inhibitors on Body Weight, BMI, and Body Composition in Adults With Type 2 Diabetes Mellitus: A Systematic Review

**DOI:** 10.7759/cureus.72771

**Published:** 2024-10-31

**Authors:** Saira Naeem, Cyprian O Ogah, Huda Mohammed, Ingie M Gabra, Nouran Halawa, Iana Malasevskaia

**Affiliations:** 1 Medicine, Faisalabad Medical University, Faisalabad, PAK; 2 Internal Medicine, Valley Baptist Hospital at University of Texas Rio Grande Valley, Harlingen, USA; 3 Colorectal Surgery, Luton and Dunstable Hospital, Luton, GBR; 4 Anesthesiology, John Muir Health, Walnut Creek Medical Centre, Walnut Creek, USA; 5 Internal Medicine, Ain Shams University, Cairo, EGY; 6 Research and Development, California Institute of Behavioral Neurosciences and Psychology, Fairfield, USA

**Keywords:** body composition, body mass index, sglt2 inhibitors, type 2 diabetes mellitus, weight management

## Abstract

Type 2 diabetes mellitus (T2DM) is a major global health issue, affecting millions and leading to significant healthcare costs. Sodium-glucose cotransporter 2 (SGLT2) inhibitors have emerged as a potential treatment option. Still, their effects on body weight, body mass index (BMI), and body composition compared to other diabetes medications or placebos remain unclear. This systematic review investigates these effects in adults with T2DM. A comprehensive literature search was conducted from June 20, 2024, to July 5, 2024, across six databases and one register: PubMed, MEDLINE, Cochrane Library (CENTRAL), Europe PMC, ScienceDirect, ClinicalTrials.gov, and EBSCO Open Dissertation, yielding 2,425 records. Following the application of inclusion and exclusion criteria, 13 studies were selected for final analysis, encompassing a sample size of 37,619 participants, adhering to Preferred Reporting Items for Systematic Review and Meta-Analysis (PRISMA) 2020 guidelines. The quality of the included studies was assessed using the Cochrane Risk-of-Bias Tool for randomized controlled trials and the Newcastle-Ottawa Scale for observational studies. Results indicate that SGLT2 inhibitors are significantly associated with reductions in body weight and BMI compared to other diabetes medications and placebo. These findings suggest that SGLT2 inhibitors improve glycemic control and facilitate effective weight management, underscoring their potential role in comprehensive diabetes care. Future research should focus on long-term outcomes and the integration of SGLT2 inhibitors into individualized treatment plans for patients with T2DM.

## Introduction and background

Type 2 diabetes mellitus (T2DM) is a critical public health concern with significant implications for individual health and healthcare costs [1]. Approximately 462 million people worldwide are affected by T2DM, accounting for 6.28% of the global population. In 2017, over 1 million deaths were attributed to this condition, making it the ninth leading cause of death [1]. Global healthcare spending related to diabetes was estimated at 966 billion USD in 2021, with projections suggesting it could rise to 1,054 billion USD by 2045. More than half a billion people worldwide are currently living with diabetes, representing over 10.5% of the global adult population [2].

The primary treatment for T2DM focuses on weight loss through lifestyle changes, which not only aids in weight management but also improves glycemic control and reduces related risk factors [3]. Intensive lifestyle interventions have been shown to lower the incidence of diabetes by 58% [4]. Research, including the Look AHEAD (Action for Health in Diabetes) study involving 5,145 participants, demonstrated that losing 5-10% of body weight can enhance fitness, lower hemoglobin A1C (HbA1c) levels, improve cardiovascular disease risk factors, and reduce the need for diabetes, hypertension, and lipid-lowering medications [5].

Despite the importance of lifestyle modifications, they often yield inconsistent results due to challenges in adherence and individual variability. Additionally, many traditional diabetes medications are linked with weight gain, which can worsen health outcomes [6]. This highlights the need for more effective therapeutic options that control blood glucose levels and facilitate weight management.

Sodium-glucose cotransporter 2 (SGLT2) inhibitors represent a new class of anti-hyperglycemic drugs approved by the FDA [7]. They lower glucose reabsorption in the renal tubules, reducing blood glucose levels without triggering insulin release [7]. Clinical trials have shown that SGLT2 inhibitors, whether used alone or in combination with other treatments, can lead to a weight loss of about one to four kg over 18 to 104 weeks [8]. Beyond their ability to lower HbA1c levels and improve metabolic control in T2DM, SGLT2 inhibitors also reduce pulmonary artery pressure in diabetic patients with heart failure, decreasing the incidence of major adverse cardiovascular events [9].

The presence of complex, systemic barriers that influence outcomes underscores the need for innovation in successfully implementing SGLT2 inhibitors for appropriate clinical indications [10]. This systematic review aims to investigate the effects of SGLT2 inhibitors on weight, body composition, and body mass index (BMI) in adults with T2DM compared to other diabetes medications or placebo.

## Review

Methods

Study Design

This systematic review was conducted following the Preferred Reporting Items for Systematic Review and Meta-Analysis (PRISMA) 2020 guidelines [11] and sought to assess how SGLT2 inhibitors influence weight, BMI, and body composition in adults with T2DM, compared to other diabetes medications or a placebo.

Eligibility Criteria

The eligibility criteria included several factors. The study involved individuals 18 years of age and above diagnosed with T2DM. Only studies published in English were considered. The study design encompassed randomized controlled trials (RCTs), cohort studies, case-control studies, and non-randomized clinical trials. The intervention focused on studies comparing SGLT2 inhibitors with other antidiabetic medications or placebo classes. Outcomes had to report changes in weight, BMI, or body composition as primary or secondary outcomes. The time frame for included studies was from January 2019 to July 2024, and only human studies were included.

Exclusion criteria were also established. Children under 18 years old, pregnant women, and breastfeeding women were excluded. Non-completed studies, studies without results, abstracts, animal studies, in vitro studies, editorials, case reports, and protocols were not considered. Additionally, studies conducted before January 2019/after July 2024 and those involving animals or non-human subjects were excluded.

Data Collection

The review searched several databases for relevant literature, including PubMed/Medline, Cochrane Library (CENTRAL), Europe PMC, Science Direct, ClinicalTrials.gov, and EBSCO Open Dissertation. A comprehensive search strategy was conducted using keywords and medical subject heading (MeSH) terms related to the population, intervention, and outcomes of interest. The search included terms associated with T2DM, SGLT2 inhibitors, and body weight outcomes, using Boolean operators to retrieve relevant studies (Table 1) comprehensively.

**Table 1:** Search strategy MeSH: medical subject heading, PMC: PubMed Central, EBSCO: Elton B. Stephens Company, CENTRAL: Cochrane Central Register of Controlled Trials, MEDLINE: Medical Literature Analysis and Retrieval System Online, RCT: randomized controlled trial

Search strategy	Databases/registers	Number of studies before and after filters	Filters applied
Type 2 diabetes"[Title/Abstract] OR T2DM[Title/Abstract] OR "non-insulin dependent diabetes"[Title/Abstract] "OR "DM type 2"[Title/Abstract] OR "T2DM"[Title/Abstract] OR "Non insulin dependent diabetes"[Title/Abstract] OR ("diabetes mellitus, type 2"[MeSH Terms] "Diabetes Mellitus, Type 2" [Mesh] AND ("SGLT2 inhibitor*"[Title/Abstract] OR "sodium-glucose co-transporter 2 inhibitor*"[Title/Abstract] OR "canagliflozin"[Title/Abstract] OR "dapagliflozin"[Title/Abstract] OR ("canagliflozin"[MeSH Terms] OR "sodium-glucose transporter 2 inhibitors"[MeSH Terms] AND ("weight"[Title/Abstract] OR "BMI"[Title/Abstract] OR "body mass index"[Title/Abstract] OR "body composition"[Title/Abstract] OR "body weight"[MeSH Terms]	PubMed/MEDLINE	2425/184	Studies included: full text, clinical study, clinical trial, observational study, RCT; time frame: for the last five years; species: humans; language: English; age: 18+ years old
#1 ("type 2 diabetes"):ti,ab,kw OR (T2DM) OR ("non-insulin-dependent NEXT diabetes mellitus"):ti,ab,kw OR (''DM type 2''):ti,ab,kw OR (“T2DM”):ti,ab,kw (Word variations have been searched) 46479 #2 MeSH descriptor: [Diabetes Mellitus, Type 2] explode all trees 26311 #3 1 OR #2 1425643 #4 ("SGLT2" NEXT inhibitor*) OR ("sodium-glucose" NEXT "co-transporter 2" NEXT inhibitor*) OR canagliflozin OR dapagliflozin OR empagliflozin OR ertugliflozin OR ipragliflozin OR luseogliflozin OR tofogliflozin 5812 #5 MeSH descriptor: [Sodium-Glucose Transporter 2 Inhibitors] explode all trees 1009 #6 #4 OR #5 5902 #7 weight OR BMI OR "body mass index" OR "body composition" OR adipos* OR obes* OR lean OR muscle OR "fat mass" OR "fat-free mass" 313614 #8 MeSH descriptor: [Weights and Measures] this term only 52 #9 MeSH descriptor: [Obesity] explode all trees 21407 #10 MeSH descriptor: [Body Mass Index] explode all trees 14064 #11 #7 OR #8 0R #9 OR #10 313614 #12 #3 AND #6 AND #11 2485	Cochrane Library (CENTRAL)	2485/1306	Language: English; time frame: last five years; studies included: clinical studies and observation studies; studies excluded: reviews, protocols, and clinical answers
“Diabetes Mellitus type 2” AND (‘’SGLT 2 inhibitor’’ OR Gliflozins OR Farxiga) AND (‘’Body weight’’ OR “weight” OR “BMI” ) AND (HAS_FT:Y) AND (((SRC:MED OR SRC:PMC OR SRC:AGR OR SRC:CBA) NOT (PUB_TYPE:"Review"))) AND (FIRST_PDATE:[2019 TO 2024])	Europe PMC	1953/901	Study design: full text, research articles; time frame: 5 years
“Diabetes Mellitus type 2” OR “Non insulin dependent diabetes mellitus” OR “DM type 2” AND ‘’SGLT 2 inhibitor’’OR Gliflozins OR Farxiga AND ‘’Body weight’’ OR “weight” OR “BMI”--Title abstract	Science Direct	3013/93	Study design: clinical study, observational study; subject area: medicine and dentistry; time frame: 5 years
“Diabetes Mellitus type 2” OR “Non insulin dependent diabetes mellitus” OR “DM type 2” OR “T2DM” OR “Non insulin dependent diabetes” AND ‘’SGLT 2 inhibitor*’’OR Gliflozins OR Flozins OR Farxiga OR Jardiance AND ‘’Body weight’’ OR weight OR BMI OR ‘’Body composition’’	ClinicalTrials.gov	22/7	Studies included: completed, interventional studies, observational studies, studies with results; age: adults 18+; language: English
(“Diabetes Mellitus type 2” OR “Non insulin dependent diabetes mellitus” OR “DM type 2” OR “T2DM” OR “Non insulin dependent diabetes” OR “Adult onset diabetes mellitus” ) AND (‘’SGLT 2 inhibitor’’OR Gliflozins OR Flozins OR Farxiga OR Jardiance OR Sodium glucose 2 inhibitor AND (“Body weight” OR “weight”OR “BMI” OR “Body composition”) NOT Review	EBSCO Open Dissertation	8/8	Study design: clinical study, full-text; time frame: 5 years; language: English

Study Selection

Two independent reviewers screened the titles and abstracts of identified studies for eligibility based on the inclusion and exclusion criteria. Full texts of potentially eligible studies were retrieved for further evaluation. Discrepancies between reviewers were resolved through discussion or consultation with a third reviewer.

Data Extraction

Data were derived from the included studies using a consistent, standardized template. The information collected included author(s) and year of publication, study design, sample size, participant characteristics (age, sex), intervention details (type of SGLT2 inhibitor, dosage), comparison group details (placebo or other diabetes medications), outcomes measured (weight, BMI, body composition), and key findings and conclusions.

Quality Assessment

The quality of the included studies was evaluated using the Newcastle-Ottawa Scale (NOS) [12] for observational studies and the Cochrane Risk-of-Bias Tool (RoB 2) [13] for RCTs. Based on the assessment criteria, studies were categorized as "good," "fair," or "poor" quality.

Data Synthesis

A descriptive synthesis was carried out to summarize the key findings of the included studies.

Results

**Figure 1:**
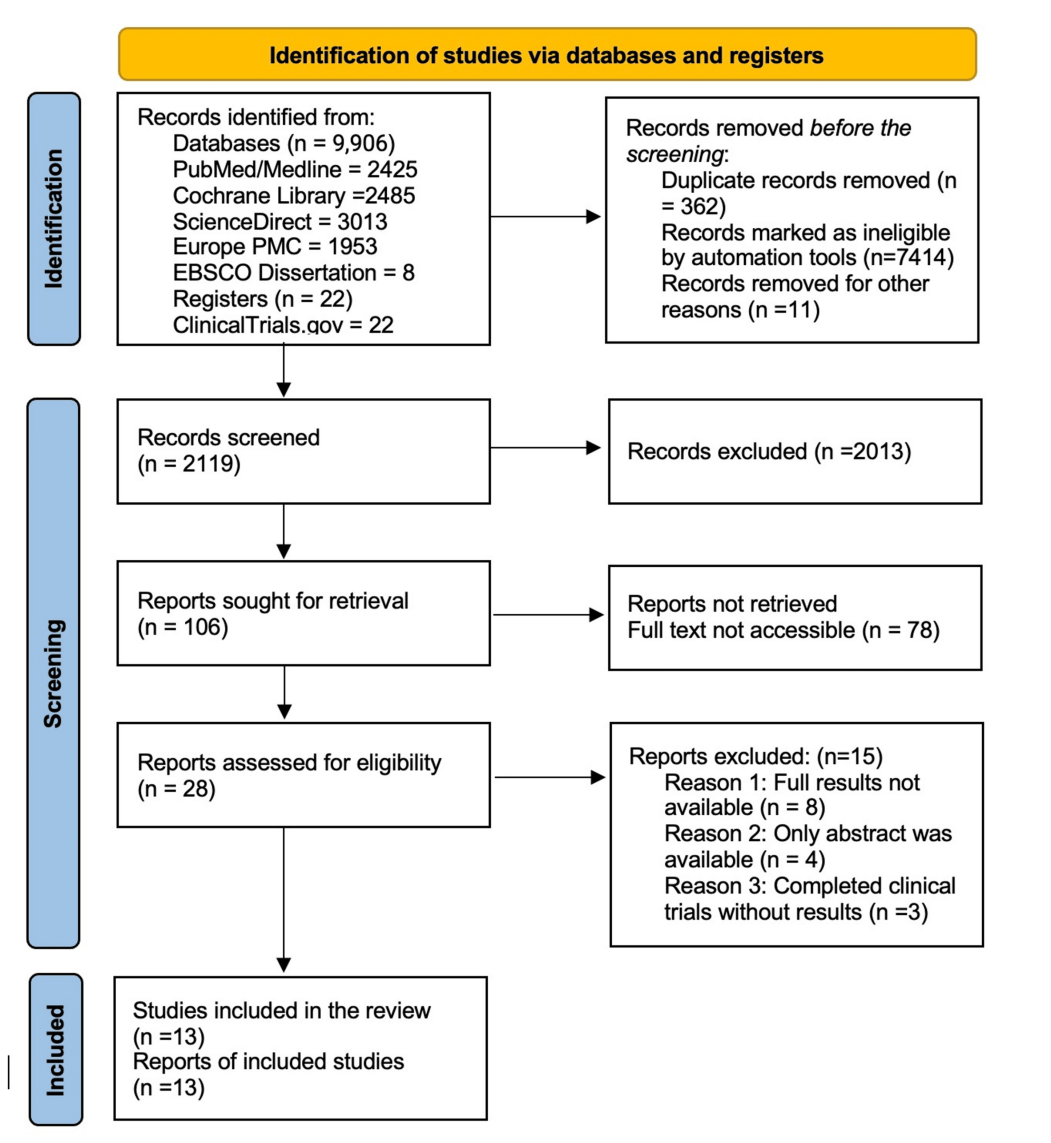
PRISMA flow diagram PRISMA: Preferred Reporting Items for Systematic Review and Meta-Analysis, MEDLINE: Medical Literature Analysis and Retrieval System Online, PMC: PubMed Central, EBSCO: Elton B. Stephens Company

A thorough search strategy outlined in the Methods section produced 9,906 initial records from multiple databases and registers. After eliminating duplicates and applying the eligibility criteria, full-text articles were selected for future evaluation. In the end, 13 studies were incorporated into the final review. The study selection process is represented in a PRISMA flow diagram (Figure 1).

Results of Risk of Bias Assessment

Table 2 provides a summary of the bias risk assessment for the included studies, which utilizes the NOS [12], converted to Agency for Healthcare Research and Quality (AHRQ) standards [14]. The assessment shows that all five studies were rated "good quality."

**Table 2:** Quality assessment of observational studies: NOS scores Note: Four stars ( * ) could be awarded for selection, two for comparability, and three for outcome domains. The total score ranges from zero to nine. The AHRQ has set quality thresholds for converting scores from the NOS. High quality: three or four stars in the selection domain, one or two stars in the comparability domain, and two or three stars in the outcome/exposure domain. Fair quality: two stars in the selection domain, one or two stars in the comparability domain, and two or three stars in the outcome/exposure domain. Poor quality: zero or one star in the selection domain, zero in the comparability domain, or zero or one star in the outcome/exposure domain. AHRQ: Agency for Healthcare Research and Quality, NOS: Newcastle-Ottawa Scale

Study/year	Selection	Comparability	Outcome	Converting the NOS to AHRQ standards
Coleman et al., 2019 [15]	* * * *	* *	* * *	Good quality
Bilgin et al., 2021 [8]	* * *	*	* * *	Good quality
Hopf et al., 2021 [16]	* * *	*	* * *	Good quality
Wang et al., 2022 [17]	* * *	* *	* * *	Good quality
Chacko et al., 2021 [18]	* * *	*	* * *	Good quality

Table 3 presents the risk of bias assessment for the included RCTs using the Cochrane RoB 2 [13]. Eight out of nine RCTs showed a low risk of bias across all domains.

**Table 3:** Risk of bias assessment of RCTs: Cochrane RoB 2 Note: RoB 2 domains: (1) randomization process, (2) deviations from planned interventions, (3) absence of outcome data, (4) outcome measurement, and (5) selection of reported result + minimal risk of bias, - significant risk of bias, ! some concerns about risk of bias RCTs: randomized controlled trials, RoB 2: Risk-of-Bias Tool

Author/year	Domain 1	Domain 2	Domain 3	Domain 4	Domain 5	Overall
Bouchi et al., 2021 [19]	+	+	+	+	+	+
Cho et al., 2019 [20]	+	+	+	+	+	+
Heymsfield et al., 2020 [21]	+	+	+	+	+	+
Vilsboll et al., 20219 [22]	+	+	+	+	+	+
Wolf et al., 2021 [23]	+	+	+	+	+	+
Inzucchi et al., 2021 [24]	+	!	+	+	+	!
Halvorsen et al., 2019 [25]	+	+	+	+	+	+
Kang et al., 2022 [26]	+	+	+	+	+	+

All studies included in the future review exhibited a low risk of bias, with none classified as having a high risk. However, Inzucchi et al. (2021) [24] indicated some concerns in Domain 2, which results in an overall assessment of some concerns regarding the risk of bias for this study (Table 3).

Summary of Included Studies

The 13 included studies primarily focus on the effectiveness and safety of various glucose-lowering agents, particularly SGLT2 inhibitors and other antidiabetic medications, in patients with T2DM. Most studies are observational or RCTs, encompassing a wide range of sample sizes, from 71 to over 20,000 participants, with ages varying from 20 to 75 years.

Common objectives across these studies include evaluating the impact of specific medications, such as canagliflozin, empagliflozin, dapagliflozin, and ertugliflozin, on crucial health parameters, including glycemic control (measured by HbA1c), body weight, blood pressure, and metabolic markers. Many studies also assess these medications' safety profiles, particularly regarding adverse events like genital infections or kidney function deterioration (Table 4).

**Table 4:** Summary of included studies RCT: randomized controlled trial, SGLT2: sodium-glucose cotransporter 2, T2DM: type 2 diabetes mellitus, DPP4: dipeptidyl peptidase-4, CHD: congestive heart disease, DAPA: dapagliflozin, PIO: pioglitazone, BEXA: bexagliflozin, ERTU: ertugliflozin, FPG: fasting plasma glucose, SAXA: saxagliptin, EMPA: empagliflozin, SBP: systolic blood pressure, GLP-1 RA: glucagon-like peptide-1 receptor agonist, HbA1c: hemoglobin A1C, BMI: body mass index

Author/year	Type of study	Sample size	Age (median/range)	Aim of study	Key findings
Coleman et al., 2019 [15]	Observational	13,612	54.2 ± 8.9/54.6 ± 13.4	Evaluate the effectiveness of canagliflozin 300 mg compared to GLP-1 RA in managing T2DM.	Patients on canagliflozin were more likely to achieve an HbA1c of less than 8.0% (p=0.0364) and a weight loss of 5% or more compared to those on GLP-1 RAs (p<0.0001).
Bilgin et al., 2021 [8]	Observational	108	58.7 ± 8.5	Examining the effects on T2DM patients treated with EMPA.	EMPA treatment showed a statistically significant average weight loss of 4 kg over six months (p<0.001). Their mean BMI showed an average reduction of 1.29 kg/m² (p<0.001) after six months and did not contribute to decreased kidney function in T2DM patients with established CHD.
Wang et al., 2022 [17]	Observational	20019		Exploring the impact of SGLT2 inhibitors on weight management in T2DM and recommending therapeutic regimens.	The study showed that various SGLT2 inhibitors require different amounts of time to reach the plateau effect on weight: 100 mg/day canagliflozin requires 13.4 weeks, 10 mg/day empagliflozin takes 67.2 weeks, 5 mg/day ertugliflozin needs 13.68 weeks, 50 mg/day ipragliflozin takes 12.36 weeks, 2.5 mg/day luseogliflozin requires 17.52 weeks, and 20 mg/day tofogliflozin takes 12.64 weeks.
Chacko et al., 2021 [18]	Observational	315	55.6/ 25 to 85	Evaluate the short-term outcomes (4 months) of EMPA use in T2DM patients, focusing on glycemic control, blood pressure, lipid levels, body weight, and adverse events.	EMPA therapy with insulin showed mean weight loss of −2.3 kg for diabetes duration <5 years and −2.1 kg for >5 years (p<0.001). Moreover, with oral agents, weight loss was −2.7 kg and −2.1 kg for <5 and > 5 years of diabetes, respectively (p<0.001).
Hopf et al., 2021 [16]	Observational	170	60.8 ± 9.7	Assess the effectiveness of SGLT2 inhibitors on metabolic parameters and patient safety in routine outpatient settings.	The study showed HbA1c decreased by −1.0% (p<0.001), body weight by −3.0 kg (p<0.001), BMI by −0.9 kg/m² (p<0.001), and systolic blood pressure by −6 mmHg (p<0.001).
Cho et al., 2019 [20]	RCT	71	63.6 ± 10.2	Compare the impact of DAPA and PIO on body weight and glycemic control.	DAPA significantly reduces HbA1c (p<0.05), promotes weight loss (p<0.01) in T2DM, and may reduce fluid retention, thereby lowering the risk of cardiovascular events.
Inzucchi et al., 2021 [24]	RCT	637	55.7 ± 9.9	Analyze how various baseline cardiometabolic risk factors influence the treatment effects of empagliflozin 10 mg and 25 mg as a second-line therapy added to metformin in patients with T2DM.	When added to metformin, empagliflozin significantly reduces HbA1c and decreases body weight, especially in patients with higher baseline levels (p<0.0001). It effectively lowers systolic blood pressure as well.
Bouchi et al., 2021 [19]	RCT	146	59 ± 10	Assess the effect of intensive exercise combined with the SGLT2 inhibitor DAPA on body composition, including fat-free mass, in patients with T2DM.	Intensive exercise does not prevent the loss of fat-free mass following SGLT2 inhibitor treatment. However, it enhances the reduction of abdominal fat, potentially leading to more significant improvements in hyperglycemia and chronic inflammation compared to DAPA alone in T2DM patients.
Kang et al., 2022 [26]	RCT	140	20-75	Comparing the glucose-lowering effectiveness, cardiometabolic effects, and safety profiles of two drugs: ipragliflozin, an SGLT2 inhibitor, and sitagliptin, a DPP4 inhibitor.	Ipragliflozin as an add-on therapy enhances glycemic control and offers vascular and metabolic benefits in T2DM patients inadequately managed with metformin and sulfonylurea. It also significantly reduces body weight, waist circumference, overall body fat mass, and abdominal visceral fat, unlike sitagliptin (all p<0.05).
Heymsfield et al., 2020 [21]	RCT	1377	57.0 ± (9.5)	Assess the effects of ERTU in overweight and obese patients with T2DM.	ERTU effectively met metabolic targets, including reducing HbA1c to below 7%, achieving weight loss of 5% or more, and lowering SBP to under 130 mmHg, compared to placebo.
Halvorsen et al., 2019 [25]	RCT	283	55.6 ± 10.6	Examine the safety and efficacy of long-term use of BEXA as a monotherapy for T2DM.	BEXA at 20 mg/day was well tolerated, leading to a sustained, clinically significant improvement in glycemic control. It also significantly reduced weight (p<0.0001) and blood pressure without an increase in significant adverse events.
Wolf et al., 2021 [23]	RCT	98	57 ± 7	Evaluated the effects of DAPA versus glibenclamide on the lean-to-total mass ratio in T2DM patients aged 40–70 with subclinical carotid atherosclerosis and HbA1c levels between 7.0% and 9.0%.	DAPA led to a reduction in total body mass (p<0.001) as well as in lean mass (p<0.001), while glibenclamide increased total body mass (p<0.001) and lean mass (p<0.001) compared to the other treatment. The lean-to-total mass ratio rose 1.2% in the DAPA group and 0.018% in the glibenclamide group (p<0.001).
Vilsboll et al., 2019 [22]	RCT	643	55.5 ± 9.6	Compared whether the oral combination of an SGLT2 inhibitor (DAPA) and a DPP4 inhibitor (SAXA) could achieve similar glycemic control to basal insulin in T2DM patients poorly controlled with metformin, without causing an increase in hypoglycemia or body weight.	The combination of DAPA + SAXA in insulin-naive patients with poorly controlled T2DM provided similar glycemic control, a lower risk of hypoglycemia, and a meaningful reduction in body weight compared to basal insulin (p<0.0001).

Discussion

SGLT2 inhibitors, or gliflozins, work by preventing glucose reabsorption in the kidney's proximal tubules [27]. This mechanism leads to a reduction in blood glucose levels and lowers HbA1c by approximately 1.0% [27]. The caloric loss associated with glucosuria promotes weight reduction, enhances insulin sensitivity, improves lipid metabolism, and may help reduce lipotoxicity [27]. Clinical trials have demonstrated that SGLT2 inhibitors significantly impact weight management in individuals with T2DM. By analyzing 13 relevant studies, we elucidated their positive effects on weight management while underscoring their cardiovascular and renal benefits.

Effects on Body Weight, Body Composition, BMI, and HbA1c

The studies reviewed encompassed a diverse population of adults aged 20 to 75 with T2DM, revealing a wide range of outcomes associated with SGLT2 inhibitors. For instance, Coleman et al. (2019) demonstrated that patients on canagliflozin achieved over 5% greater weight loss compared to those on GLP-1 receptor agonists, alongside significant reductions in HbA1c, with a higher likelihood of reaching HbA1c levels below 8.0% [15]. Similarly, Bilgin et al. (2021) reported an average weight loss of 4 kg and a BMI reduction of 1.29 kg/m² in patients treated with empagliflozin over six months, reinforcing the efficacy of this medication in managing both weight and glycemic control [8].

Wang et al. (2022) provided a unique perspective by investigating the time to reach a weight loss plateau across various SGLT2 inhibitors, revealing that canagliflozin required 13.4 weeks, while empagliflozin took significantly longer at 67.2 weeks [17]. This highlights the variability in response times among agents, which is crucial for clinicians when considering treatment options.

Hopf et al. (2021) further supported these findings, reporting a 3 kg weight loss, a 0.9 kg/m² decrease in BMI, and a 1.0% reduction in HbA1c among participants using SGLT2 inhibitors [16]. Moreover, Heymsfield et al. (2020) indicated that ertugliflozin effectively met critical metabolic targets, achieving HbA1c levels below 7% and significant weight loss, emphasizing its role in comprehensive diabetes management [21].

Halvorsen et al. (2019) demonstrated that the SGLT2 inhibitor bexagliflozin effectively lowers HbA1c and significantly reduces weight. The active group showed a mean weight loss of −2.63 kg at week 24 and −2.41 kg at week 96 from baseline [25].

Comparison of SGLT2 Inhibitors With Other Antidiabetic Medications

The comparative effectiveness of SGLT2 inhibitors versus other antidiabetic medications was also notable in our review. Cho et al. (2019) found that dapagliflozin not only achieved a modest reduction in HbA1c but also significantly reduced body weight compared to pioglitazone while lowering the risk of fluid retention and subsequent cardiovascular events [20]. This contrast underscores the distinct advantages of SGLT2 inhibitors in managing glycemic control and weight.

Wolf et al. (2021) compared the effects of dapagliflozin and glibenclamide on weight, finding that dapagliflozin significantly reduced both total body mass and lean mass (p<0.001). In contrast, glibenclamide increased total body and lean mass (p<0.001) relative to the other treatment. Additionally, the lean-to-total mass ratio increased by 1.2% in the dapagliflozin group and by 0.018% in the glibenclamide group [23].

Vilsboll et al. (2019) found that combining dapagliflozin and saxagliptin significantly reduced body weight in insulin-naive T2DM patients while maintaining similar glycemic control and a lower hypoglycemia risk compared to basal insulin [22].

Use of SGLT2 Inhibitors in Combination With Other Antidiabetic Medications

Combining SGLT2 inhibitors with other antidiabetic therapies enhances their effectiveness. Chacko et al. (2021) reported that patients receiving SGLT2 inhibitors alongside insulin experienced weight loss of 2.3 kg for those with diabetes duration under five years and 2.1 kg for those with longer durations [18]. Similarly, Inzucchi et al. (2021) demonstrated that adding empagliflozin to metformin significantly lowered HbA1c and body weight, particularly in patients with higher baseline BMIs [24]. Kang et al. (2022) highlighted the advantages of Ipragliflozin as an adjunct treatment, reporting better glycemic control and reductions in body weight and abdominal fat compared to sitagliptin [26].

Combined Effects of SGLT2 Inhibitors and Lifestyle Modifications

Lastly, the interplay between SGLT2 inhibitors and lifestyle modifications warrants attention. Bouchi et al. (2021) found that while intensive exercise did not prevent the loss of fat-free mass during SGLT2 inhibitor therapy, it significantly enhanced abdominal fat reduction, potentially leading to better management of hyperglycemia and chronic inflammation compared to dapagliflozin alone [19]. This finding suggests that a multifaceted approach, combining pharmacological and lifestyle interventions, may yield superior outcomes for patients with T2DM.

Other Beneficial Effects of SGLT2 Inhibitors

Studies have shown that beyond their positive effects on HbA1c, body weight, body composition, and BMI, SGLT2 inhibitors also provide significant benefits for blood pressure, renal function, and cardiovascular health. Heymsfield et al. (2020) demonstrated that ertugliflozin effectively lowers blood pressure to below 130 mmHg compared to placebo [21]. Inzucchi et al. (2021) found that empagliflozin significantly reduces systolic blood pressure at both 10 mg and 25 mg doses combined with metformin [24]. Chacko et al. (2021) highlighted that empagliflozin not only improves cardiovascular outcomes by reducing the risk of cardiac events but also leads to slight reductions in total cholesterol, triglycerides, and low-density lipoprotein cholesterol, with a slight increase in high-density lipoprotein cholesterol after four months of treatment in both insulin and oral agent combination groups [18]. Hopf et al. (2021) demonstrated improved kidney function, evidenced by a 7.0 mL/min increase in estimated glomerular filtration rate and a reduction in systolic blood pressure by 6 mmHg [16].

Clinical Implications

This systematic review's findings emphasize the critical role of SGLT2 inhibitors in controlling body weight among individuals with T2DM. Clinicians should consider these medications for their glycemic control benefits and potential to facilitate weight loss and improve overall metabolic health. Given the established link between weight management and enhanced glycemic control, incorporating SGLT2 inhibitors into treatment plans could be particularly beneficial for overweight or obese patients. Additionally, these agents' favorable safety profile and cardiovascular benefits support their use in a broader range of diabetic patients, especially those with comorbid conditions such as heart disease.

However, healthcare providers must individualize treatment plans based on patient characteristics, including baseline body weight, age, and comorbidities. Regular monitoring of weight and metabolic parameters should be integrated into routine care to assess the effectiveness of SGLT2 inhibitors and make necessary adjustments. Furthermore, patient education regarding the importance of lifestyle modifications and pharmacotherapy remains crucial for optimizing outcomes. Overall, the integration of SGLT2 inhibitors into diabetes management strategies presents a promising avenue for improving both glucose control and weight management in those with T2DM.

Strengths and Limitations of the Review and Included Research Studies

The review presents several strengths that enhance its credibility and relevance in examining the effects of SGLT2 inhibitors on body weight in patients with T2DM. One notable strength is the comprehensive search strategy, which involves multiple databases. This extensive approach ensures a wide-ranging collection of relevant studies, enhancing the findings' robustness. Additionally, including diverse study designs, such as RCTs and observational studies, allows for a more comprehensive understanding of the effectiveness and safety of SGLT2 inhibitors across different populations and clinical settings.

Another significant advantage is the focus on recent data, with the review concentrating on studies published within the last five years. This emphasis on current research is crucial, given the rapid advancements in diabetes treatments and the evolving understanding of SGLT2 inhibitors. Furthermore, the review's application of established quality assessment tools, namely NOS for observational studies and the Cochrane RoB 2 for RCTs, adds credibility by ensuring that the included studies adhere to recognized methodological standards. By specifically targeting the impact of SGLT2 inhibitors on body weight, the review addresses a critical aspect of diabetes management, linking weight control to improved metabolic outcomes.

However, the review has its limitations. One primary concern is the heterogeneity of the included studies, which varied in design, sample size, and participant characteristics. This variability may introduce inconsistencies in results and complicate the synthesis of findings. Moreover, while the studies provide valuable insights into short- to medium-term effects, long-term follow-up data are needed. Such data is essential for assessing the sustainability of weight loss and other health benefits associated with SGLT2 inhibitors.

Additionally, the observational studies included in the review are susceptible to biases, such as confounding factors and selection bias, which can affect the reliability of their results compared to those from RCTs. The review's exclusion of non-English studies further limits its scope, as it may overlook relevant research published in other languages, affecting the findings' generalizability. Lastly, the variability in outcome measures across studies, particularly in how body weight and metabolic parameters are assessed, could lead to inconsistencies in reported outcomes, complicating comparisons across studies. Furthermore, the reliance on published studies raises the potential for publication bias, as studies with negative or inconclusive results are less likely to be disseminated, potentially skewing the overall findings.

While the review provides valuable insights into the role of SGLT2 inhibitors in weight management for people with T2DM, it is vital to fully consider these strengths and limitations to understand the findings' implications.

Future Research Directions

Future research should address several gaps identified in the current literature on SGLT2 inhibitors and weight management. Long-term research is essential to determine the lasting effects of weight loss and metabolic improvements associated with these medications over extended durations. Investigating the impact of SGLT2 inhibitors in diverse populations, including different age groups and ethnicities, will broaden the applicability of findings and ensure that treatment recommendations are relevant to a broader range of patients.

Further exploration into the mechanisms by which SGLT2 inhibitors influence weight loss could provide valuable insights into optimizing their use. Comparative studies assessing the efficacy of SGLT2 inhibitors against other classes of antidiabetic medications, particularly in weight management, would also be beneficial. Additionally, research should focus on understanding the impact of SGLT2 inhibitors on body composition beyond simple weight metrics, such as fat mass and lean body mass, to provide a more comprehensive picture of their effects.

Finally, qualitative studies exploring patient experiences and perceptions regarding SGLT2 inhibitors and weight management could offer valuable insights into adherence and treatment satisfaction. By addressing these areas, future research can contribute to a more nuanced understanding of SGLT2 inhibitors' role in diabetes care and inform clinical practice guidelines.

## Conclusions

This systematic review provides compelling evidence that SGLT2 inhibitors significantly impact weight, BMI, and body composition in adults with T2DM. The findings highlight the critical role of incorporating SGLT2 inhibitors into diabetes management protocols, emphasizing their dual benefits in both glycemic control and supporting weight loss while improving overall metabolic health. Given the rising prevalence of obesity among individuals with T2DM, these medications offer a dual benefit that can enhance patient outcomes. Future research should focus on long-term effects and the optimal integration of SGLT2 inhibitors into individualized treatment plans, considering patient-specific factors such as baseline weight and comorbidities. Overall, the evidence supports the expanded use of SGLT2 inhibitors as a valuable component in managing T2DM and associated weight challenges. Furthermore, SGLT2 inhibitors are effective in reducing blood pressure, preventing renal damage in T2DM, and improving cardiovascular outcomes.
